# Analysis of single cell data as it relates to aging and longevity

**DOI:** 10.1093/geroni/igab046.2539

**Published:** 2021-12-17

**Authors:** Tanya Karagiannis, Todd Dowrey, Carlos Villacorta-Martin, George Murphy, Stefano Monti, Paola Sebastiani

**Affiliations:** 1 Tufts Medical Center; 2 Boston University, Boston, Massachusetts, United States; 3 Boston University School of Medicine, Boston, Massachusetts, United States; 4 Tufts Medical Center, Physician Organization, BOSTON, Massachusetts, United States

## Abstract

Age-related disability and diseases are known to be delayed in people living to 100 years or more. Changes in the immune system with age are known, including in cell type composition and gene expression differences. To further explore changes in extreme longevity subjects, we investigated peripheral blood immune system cell subpopulations across age and extreme longevity at a single cell resolution. We performed an integrative analysis of public scRNA-seq datasets to define consensus cell types of longevity and age, and classified cell types in our novel New England Centenarian Study dataset. We integrated these datasets together to investigate cell type specific differences at a composition and gene expression level. Our findings identified higher cell type diversity in extreme longevity subjects compared to younger age groups, but no significant difference among younger age groups demonstrating that overall composition differences are unique to longevity. We identified novel differences in myeloid and lymphocyte populations; Extreme longevity subjects have higher composition of CD14+ Monocytes, Natural Killer cells, and T gamma delta populations and lower composition of CD16+ Monocytes and dendritic populations. We characterized gene expression differences between extreme longevity and younger age groups and differences in aging across younger age groups. We found that extreme longevity cell type specific signatures overlapped with the aging signatures by at least 50%. We identified unique genes to extreme longevity that are enriched for pathways specific to immune activation and inflammation, suggesting a protective mechanism for centenarians through efficient activation and regulation of immune subpopulations in peripheral blood.

